# Validation Study of Airgo, an Innovative Device to Screen Sleep Respiratory Disorders

**DOI:** 10.3389/fmed.2022.938542

**Published:** 2022-07-01

**Authors:** Alberto Braghiroli, David Kuller, Massimo Godio, Fabio Rossato, Carlo Sacco, Elisa Morrone

**Affiliations:** ^1^Sleep Lab, IRCCS, Veruno, Italy; ^2^Department of Pulmonary Rehabilitation, Salvatore Maugeri Foundation, Gattico-Veruno, Italy; ^3^Myair Inc., Boston, MA, United States; ^4^Myairgo Italy Srl, Milan, Italy

**Keywords:** respiratory pattern detection, respiratory sleep disorders, screening, sleep apnea, wearable devices and sensors

## Abstract

**Background:**

Obstructive sleep apnea affects a consistent percentage of the population, and only a minority of patients have been diagnosed and treated because of a discrepancy between resources available for diagnosis and the epidemiology of a disorder possibly affecting nearly one billion people in the world.

**Aim:**

We conducted a study to compare a standard home respiratory monitoring system (Nox T3) with a novel device (Airgo™) consisting of an elastic band and a small recorder, light, comfortable for the patient, and low-cost complete with automatic analysis of the data that produces a screening report indicating the type and severity of sleep respiratory disorder.

**Patients and Results:**

We examined 120 patients, reduced to 118 for technical problems. The mean (*SD*) age of the patients is 55.7 ± 13 years, their BMI is 27.8 ± 4.3 kg/m^2^, and their AHI is 22 ± 22 events/h. Patients belong to all the different severity rates of OSA, with a percentage of them classified as free of respiratory disorders. The Airgo™ showed excellent agreement with the results of the gold standard, reporting high levels of sensitivity, specificity, positive and negative predicted value, and accuracy.

**Conclusion:**

Airgo™ is a reliable tool to screen patients with suspected sleep respiratory disorders, well tolerated by the patient based on totally automatic analysis and reporting system, leading to more efficient use of doctor’s and clinician’s time and resources and extending the opportunity to diagnose more possible candidates for treatment.

## Introduction

Sleep respiratory disorders are a common problem in the general population (1). Obstructive sleep apnea (OSA), a recurrent collapse of the upper airways occurring during sleep, leads to sleep fragmentation and intermittent oxyhemoglobin desaturations, which are significant risk factors for cardiovascular, cerebrovascular, and metabolic disorders (2). Since the disorder occurs only during sleep, patients are usually unaware of their condition and have to rely on symptoms that are not related to upper airway function or to the respiratory system (i.e., nocturia, daytime fatigue, somnolence) or on somebody who witnesses the respiratory events during sleep and suggests seeking medical advice. A proper diagnosis is often delayed until after severe consequences have occurred (3).

Guidelines on the diagnosis of OSA (4) require an overnight study with polysomnography, a complex and expensive supervised monitoring of several vital functions, or at least a polygraphy, including a more limited set of sensors easy to apply at home. Considering the epidemiology of OSA (1), this is a considerable bottleneck in the management of patients since the estimate of people affected worldwide is close to one billion (5). Screening tools have been proposed based on a more limited set of sensors (i.e., oximetry) or questionnaires combining symptoms and clinical signs to identify subjects at risk. Excessive daytime somnolence is a landmark symptom easily measured with self-administered scales (Epworth sleepiness scale, Stanford sleepiness scale) (6), but it occurs only in one out of five patients with OSA (1). Similarly, oximetry is unreliable in detecting moderate OSA in patients with short events and good baseline SpO_2_ values (7).

Common disorders like cardiac arrhythmias, mainly atrial fibrillation, or diabetes, stroke, chronic heart failure, and drug-resistant hypertension show a high OSA prevalence (up to 80% in paroxysmal atrial fibrillation) (8). In these patients, screening strategies currently available do not detect patients with OSA since they do not report daytime symptoms, and the employment of current diagnostic procedures extensively is an unreliable strategy considering epidemiology and the need for a timely diagnosis (9, 10).

A simple to use, reliable, and cost-effective screening device to rule-in or rule-out the occurrence of OSA would be a significant step forward in the management of the disease. The aim of the present study is to assess the performance of Airgo™, an innovative system of respiratory monitoring, as a screening tool for respiratory sleep disorders.

## Materials and Methods

### Study Design

The study is a single center, prospective comparison of a new device to assess sleep respiratory disorders vs. standard cardiorespiratory monitoring. Consecutive patients presenting at the sleep lab of Salvatore Maugeri IRCCS Veruno Medical Center were enrolled irrespective of the type of referred symptoms. Age under 18 years, pregnancy and inability to sign informed consent were the exclusion criteria. The study was approved by the “Salvatore Maugeri Foundation” ethics committee (2300 CE) and all the patients signed informed consent.

### Devices

**FIGURE 1:**
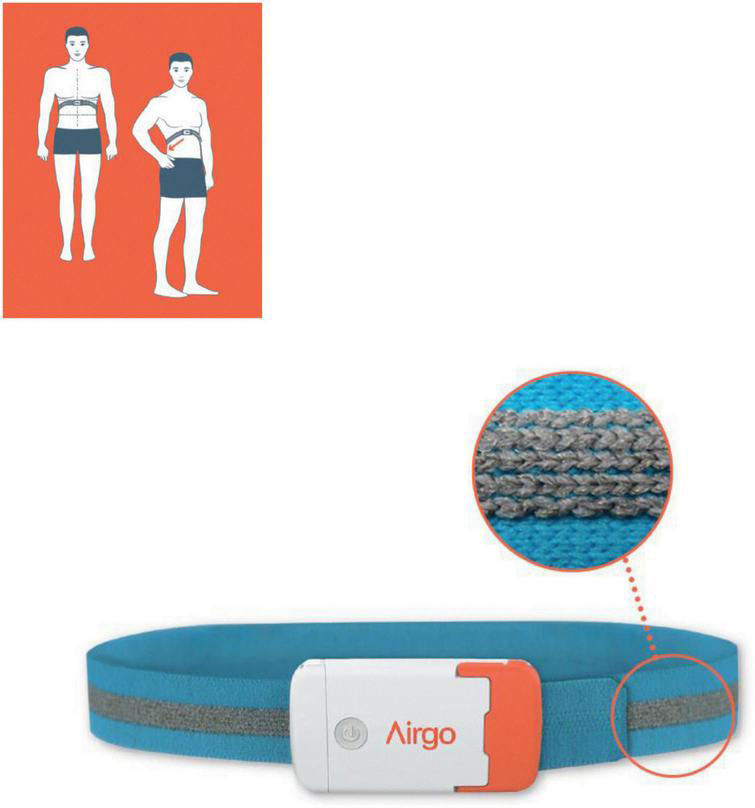
The wearable device consists of an elastic band incorporating a silver-coated electrically conductive yarn (see detail), coupled with a microprocessor embedded in an ABS shell. The figure shows the proper placement position.

**FIGURE 2:**
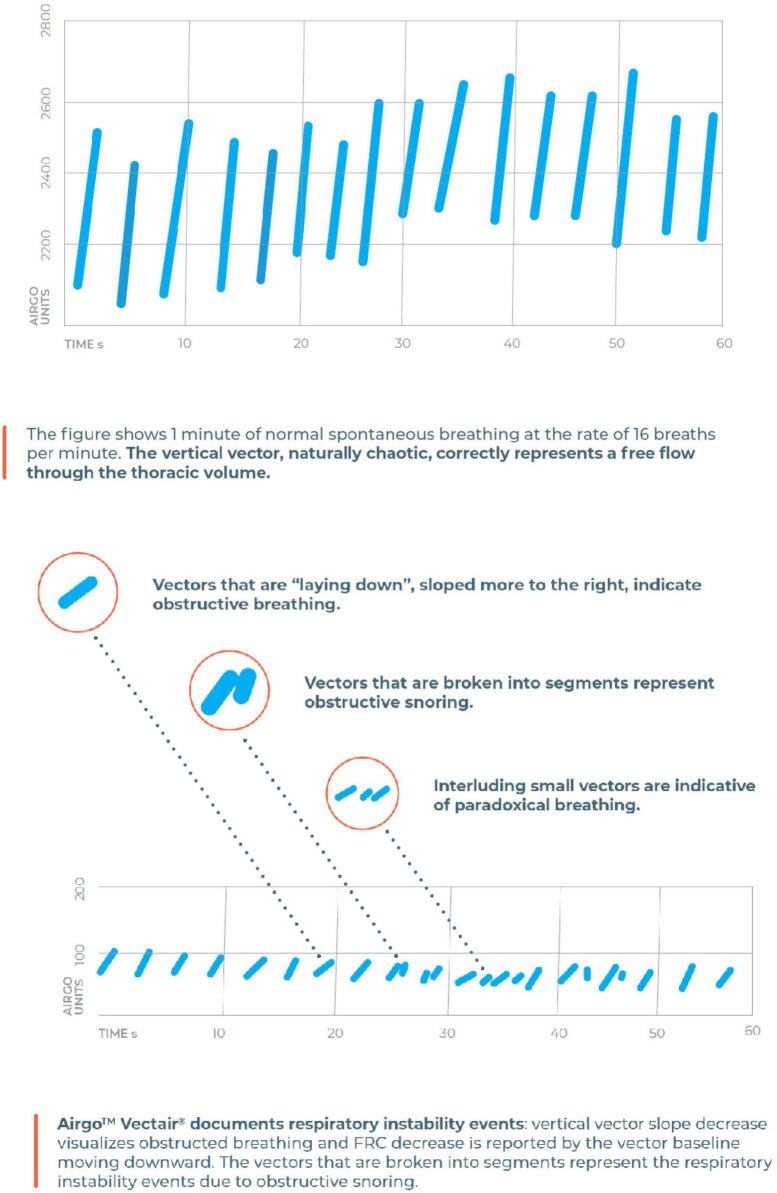
The algorithm of Airgo^™^ transforms every breath into a vector with a specific length (corresponding to tidal volume), a baseline (corresponding to the punctual functional residual capacity) and a shape here described as proportional to upper airway patency.

**FIGURE 3:**
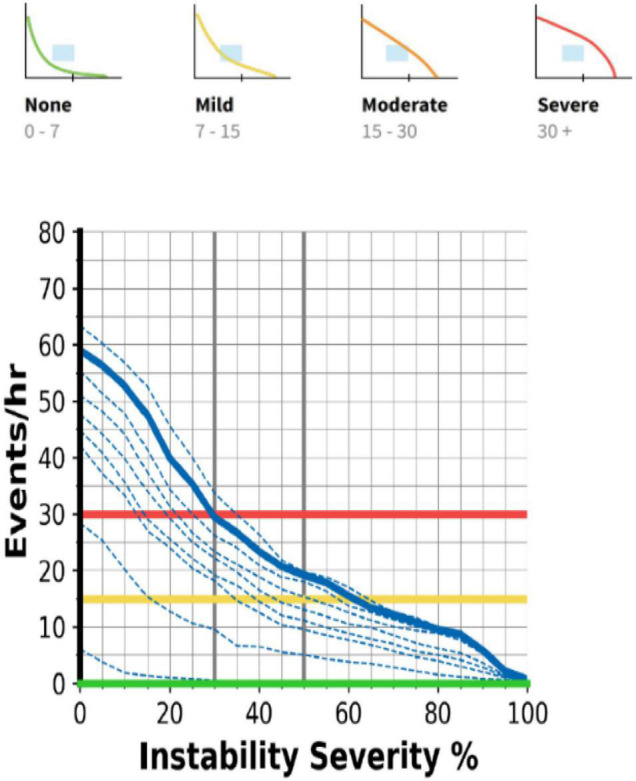
The respiratory instability curves (RIC) discriminate the occurrence, severity, and type of sleep respiratory disorder according to the interception of the target area. See text for details.

Airgo™ (Myair Inc., Boston, MA, United States; Myairgo Italy Srl, Milan, Italy) is a wearable device consisting of an elastic band incorporating a silver-coated electrically conductive yarn, coupled with a microprocessor embedded in an ABS shell (Figure 1). The band is positioned at the level of the floating ribs and monitors continuously the changes in the volume of the thoracic cage by tracking the variation in electrical resistance of the silver-coated yarn as the circumference varies. The procedure of signal reconstruction and respiratory pattern assessment has been reported extensively previously (11). Briefly, the device samples data at a frequency of 10 Hz and identifies for each breathing cycle a minimum and a maximum. The patented algorithm produces a vector connecting these two points, which allows the computation of tidal volume and respiratory rate. Vectors are a very informative signal since their baseline points correspond to the functional residual capacity of every breath, their lengths allow the calculation of tidal volumes in arbitrary units and the slopes of the vectors or their fragmentation are indicative of the coordination between thorax and abdomen. Every time an upper airway obstruction occurs, there is a paradoxical movement (partial or complete according to the degree of obstruction) of the thoracic cage, which changes the slope and the shape of the vectors as shown in Figure 2. The changes in minute ventilation are calculated dynamically by comparing the window of the last 10 s (MV10) with the mean of the previous 60 s (MV60) and respiratory events are detected when MV10 crosses the threshold of MV60. The breath by breath assessment facilitates the construction of respiratory instability curves (RICs), a very informative visualization of respiratory patterns during sleep since they do not focus on respiratory events *per se*, but on the variability of respiratory amplitude, which becomes apparent when comparing the deciles of MV reduction. The trend of RICs is thus informative on the occurrence of respiratory events as well as respiratory instability, which could be of non-respiratory origin (e.g., periodic leg movements, insomnia) (Figure 3).

The device includes three accelerometers to assess body position. This is particularly useful for correlating the occurrence and severity of respiratory events with body position. Although actigraphy has specific rules which cannot be fulfilled by a device positioned on the thorax, the signal is processed to describe both the position and activity of the patient, which easily identifies when the patient wakes up and gives information useful to discriminate wakefulness during the recording.

Nox T3 (Nox Medical, Reykjavík Iceland) has been used as the gold standard in a setting fulfilling the recommendations for home cardiorespiratory sleep studies, including nasal cannula, thoracic and abdominal inductive plethysmographic bands, pulse oximetry, microphone for snoring, body position sensor, and leg movement sensors.

Patients came to the lab in the afternoon and were instructed on how to wear the sensors of Nox T3 at home. Airgo™ was positioned by the sleep technician, and the patient was instructed to leave it as placed. A telephone line with the hospital was available in case of problems. Synchronization of the two devices was set with an iPhone used to manage the starting procedures of the Airgo™, and the same time was set on the PC at the initialization of Nox T3.

### Event Detection

Data from cardiorespiratory monitoring were manually scored in accordance with the AASM 2012 recommendations for adults (12). Apnea has been defined as the cessation of airflow for at least 10 s; hypopnea is the reduction of airflow signal of at least 30% with an oxyhemoglobin desaturation of 3% or more.

**FIGURE 4:**
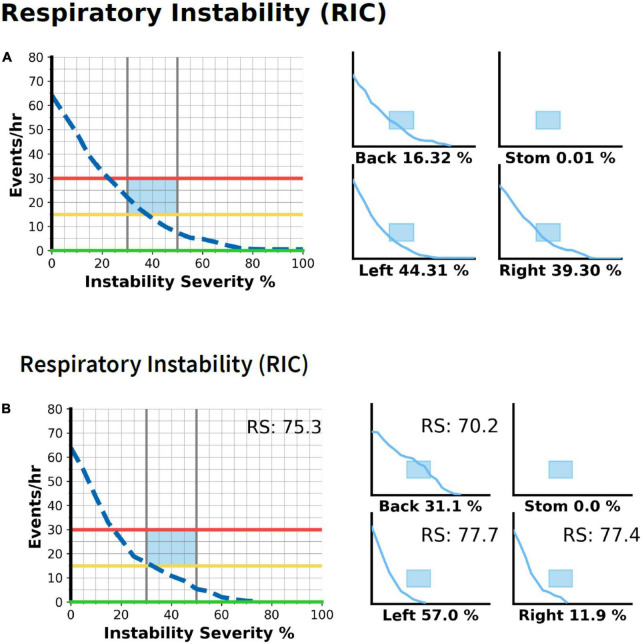
The postural OSA is detected by comparing RICs in different body positions. On the left is the whole night RIC graph, on the right data are split by body position, with the percentage of time spent in every position reported. **(A)** The patient is classified as mild-to-moderate OSA and events occur irrespective of body position. **(B)** The patient would be classified as mild OSA considering the graph of the whole night, instead, he has severe sleep apnea in the supine position and almost no events in the lateral position.

Airgo™ does not record oxyhemoglobin saturation and relies on a complex algorithm to detect the changes in tidal volume and minute ventilation. Central apnea is easily detected by the absence of the vectorized signal. Obstructive events are detected by a reduction in minute ventilation and a change in the morphology of the vectors caused by paradoxical movements of the thoracic cage. Since the desaturation criterion cannot be applied to validate hypopneas, small changes in ventilation could lead to false-positive detections. Considering that the aim of the study is to assess the reliability of Airgo™ as a screening tool for respiratory sleep disorders, a graphic interpretation of the RICs automatically generated by the program has been developed to describe the pattern and severity of the events detected (Figure 4). Patients are allocated to one of the following groups: no respiratory events; mild-to-moderate sleep apnea (AHI 5–30 at the Nox study); severe sleep apnea (AHI 30 or more at the Nox study); positional sleep apnea (pOSA); respiratory pattern instability. The latter group is a miscellanea of sleep disturbances of non-respiratory origin (i.e., myoclonus, insomnia), or occurring only in specific sleep stages (i.e., REM-related OSA), which cause a quick change in the trend of the RIC deciles.

### Statistical Analysis

Quantitative variables are expressed as the mean ± *SD*. Qualitative variables are expressed as the number of patients and percentage. Positive predictive value, negative predictive value, sensitivity, specificity, positive likelihood ratio, negative likelihood ratio, and accuracy have been calculated for every category of disease detected by Airgo™ compared with Nox results, with 95% confidence intervals for every category.

## Results

One hundred twenty patients (23 F) agreed to participate in the study. The mean (*SD*) age of the patients is 55.7 ± 13 years, their BMI is 27.8 ± 4.3 kg/m^2^, and their AHI is 22 ± 22 events/h. Two patients were excluded from the analysis, one who inadvertently switched off the Airgo™ before the beginning of recording, other for the loss of the oximetry sensor of the Nox T3 for more than 70% of the night, and refusing to repeat the study. Table 1 reports anthropometric data for the different groups. We recruited consecutive patients presenting to our sleep center, so the woman:man ratio was 1:5, a common bias of sleep centers case series compared to the prevalence of the disorder in the global population. Airgo™ classified properly 27 severe OSA pts, 16 postural OSA (pOSA), 16/19 non-OSA pts (3 FP), 35/40 mild-to-moderate OSA (3 scored severe and 2 FN), and 14/16 pts (2 FN) with irregular breathing of non-OSA origin (Table 2). The diagnostic accuracy evaluation is shown in Table 3 reporting data on the whole population studied and in Table 4 reporting data on patients with OSA or normal breathing, excluding patients with irregular breathing of non-OSA origin to focus on the performance of the device in the OSA group. In Table 5 the time spent on the single steps of the recording procedure has been compared for the two systems.

**TABLE 1:** Anthropometric data and results of sleep studies in the different groups of patients (mean ± *SD*).

	Normal	Mild-to-moderate OSA	Severe OSA	Positional OSA	Non-OSA disease
Nr	19	40	27	16	16
Age	49.8 ± 14.14	56.7 ± 10.3	59.5 ± 14.4	56.2 ± 11.1	56.1 ± 10.2
Height (cm)	171.3 ± 9.7	171.8 ± 9.9	172.8 ± 6.5	173.2 ± 9.9	170.4 ± 9.2
Weight (Kg)	76.9 ± 12.9	81.5 ± 14.7	89.5 ± 13.7	78.4 ± 13.6	79.4 ± 13.7
BMI (Kg/m^2^)	26.2 ± 3.6	27.6 ± 4.1	30.0 ± 4.6*	26.1 ± 4.0	27.3 ± 4.0
AHI	2.5 ± 1.4	15.2 ± 6.3	57.2 ± 15.2	14.2 ± 8.9	13.1 ± 9.1

*P < 0.05.

**TABLE 2:** Comparison of diagnosis obtained with Airgo^™^ vs. Nox T3 (confusion matrix).

		Nox T3				
		
Airgo™		Normal	Mild-to-moderate OSA	Severe OSA	Positional OSA	Non-OSA disease
	Normal	16	2	0	0	2
	Mild-to-moderate OSA	3	35	0	0	0
	Severe OSA	0	3	27	0	0
	Positional OSA	0	0	0	16	0
	Non-OSA disease	0	0	0	0	14

**TABLE 3:** Evaluation of Airgo^™^ performance vs. Nox T3 in classifying OSA occurrence (*n* = 118).

	Value	95% CI
Prevalence (%)	70.3	62.1–78.5
Sensitivity (%)	97.6	94.8–1.0
Specificity (%)	91.4	86.3–96.5
Positive predictive value (%)	96.4	93.0–99.8
Negative predictive value (%)	94.1	89.8–98.3
Positive likelihood ratio	11.4	5.7–17.1
Negative likelihood ratio	0.03	−0.01–0.06
Accuracy (%)	95.8	92.2–99.4

*CI, confidence interval.*

**TABLE 4:** Evaluation of Airgo^™^ performance vs. Nox T3 in classifying OSA severity (*n* = 102).

	Value	95% CI
Prevalence (%)	78.4	70.4–86.4
Sensitivity (%)	97.5	94.4–1.0
Specificity (%)	72.7	64.1–81.3
Positive predicted value (%)	92.8	87.8–97.8
Negative predicted value (%)	88.8	82.7–94.9
Positive likelihood ratio	3.6	−0.0–0.07
Negative likelihood ratio	0.03	−0.01–0.01
Accuracy (%)	92.1	86.9–97.3

*CI, confidence interval.*

**TABLE 5:** Comparison of time spent on the standard procedures to record sleep studies and produce a final report.

	Airgo	Nox T3
Device preparation	5–10	15–20
Patient training to wear the device	3–12	15–30
Pre-scoring procedures	5–10	10
Scoring of events by experts	0	60–120
Report production	5	5
Cleaning	2	5–10
Total	20–39	110–195

*Data are expressed in minutes.*

## Discussion

Our study shows that Airgo™ can be used to screen for sleep respiratory disorders with an easy-to-use, non-invasive device and without the need for manual analysis. The trend of RICs is an informative and innovative modality to rule in and rule out the occurrence of respiratory sleep disorders and immediately identifies the more severe OSA patients (i.e., AHI > 30 events per hour), those who have respiratory events only or mostly in the supine position (p-OSA) and patients with complex respiratory disorders who will probably benefit from formal polysomnography to define a proper diagnosis.

In our case series, 27 patients (23% of the sample), all males, had severe obstructive sleep apnea. Airgo™ was identified correctly in 100% of the cases. This could be very useful in clinical practice in several settings. Severe OSA has three landmarks, namely, recurrent desaturations, which cause an increase in the production and release of inflammatory factors; increased ortosympathetic tone, which is caused by autonomic nervous system activation and the release of epinephrine; a mechanical stretch of the heart walls, which causes nocturia and eases the insurgence of arrhythmias; and eccentric ventricular hypertrophy (8). The combination of these three factors in populations already predisposed to cardiovascular disease poses an additional risk of events. In the DREAM study (13), consecutive patients with an indication of PM had a prevalence of moderate to severe OSA assessed with a PSG of 78%. In paroxysmal atrial fibrillation, OSA can occur in up to 80% of patients. Most of these subjects do not report the classic symptoms of OSA patients, particularly do not complain of excessive daytime sleepiness (EDS). In the Hypnolaus study (1), a large population-based, epidemiologic study conducted in Lausanne, Switzerland, only one out of five patients with OSA complained of EDS. Therefore, although EDS is often the symptom prompting the patient to look for medical advice, in comorbid populations, particularly in cardiovascular patients, many patients do not report its occurrence. In these subjects, there is a clear need for a device that objectively measures and identifies the disorder and can be applied to large populations. Standard diagnostic procedures based on home sleep studies are currently insufficient to deal with the number of patients affected by a treatable disease, such as drug-resistant hypertension or a non-dipping profile, drug-resistant diabetes, cardiac arrhythmias, and stroke. Airgo™ could be proposed as a possible tool since automatic analysis requires no operator time to immediately identify patients with severe OSA. Although this is just a screening and does not properly classify the degree of the global risk of the patient since no information on the depth of desaturations can be obtained, it could nonetheless reliably identify the most severe patients in a case series of consecutive patients at high-risk. This could be used to prioritize waiting lists for home sleep studies, predict appropriateness in using less complex devices than polysomnography, identify subjects at risk of primary intervention failure, such as ablation for atrial fibrillation, or identify subjects at increased anesthesiologic risk for surgery patients.

Respiratory data are correlated with the signal of the three accelerometers indicating the position, and a RIC analysis is available for every position. This is crucial to identify patients with pOSA (14), 16 in our case series, who have all been identified correctly. A prevalence of 13% of pOSA is close to figures of the literature. Considering that pOSA can be treated effectively with specific instruments (15), such as postural trainers or mandibular advancement devices, when waiting lists for standard diagnosis cause a significant delay in the management, the treatment could be instituted immediately. The percentage of time spent in the different positions is reported under each RIC, and if less than 10% of time is spent in the supine position, the reliability is low and requires further validation. Night to night variability is common in pOSA and mean AHI can vary substantially, but the index in the supine position is quite constant and its severity is informative to the clinician on the opportunity to begin treatment.

A screening tool is particularly useful when it correctly identifies subjects who do not have the disease. In our case series, 16 patients had an AHI below 5, and Airgo™ identified properly 14 patients. The other two patients were misclassified as having moderate OSA since they had an intermittent flow limitation mimicking hypopneas, but without 3% desaturation. As a consequence, they had fluctuations of ventilation greater than 30%, but the high basal saturation and the short length of events did not cause significant desaturations. The definition of hypopnea has been revised in the guidelines several times, and the last edition of AASM recommendations has privileged the link to a desaturation of at least 3% to validate the semi-quantitative assessment of ventilation provided by nasal cannula and respiratory bands (16). This approach has a limit compared to the polysomnographic definition of earlier editions, which included an arousal at the end of the event even without desaturation (16). An arousal increases the tidal volume since respiratory control shifts from sleep drive to wakefulness drive (17), which is set on a lower level of carbon dioxide. Airgo™ relies only on changes in ventilation to detect respiratory events, so compared to home sleep studies, it can overestimate the severity of the disorder in some patients, particularly when wakefulness respiratory drive is more active and causes big breaths at airflow resumption.

Considering the aim of screening, the correct identification of 87% of patients without OSA is important to avoid worthless polygraphy/polysomnography, sparing resources, and shortening waiting lists. The false-positive patients (13% in our case series) have no harm since they will undergo a home sleep study that will show no respiratory events requiring treatment.

Mild-to-moderate OSA patients require a formal sleep study for a proper diagnosis. The correct identification of this group in the screening could be assigned a lower priority in waiting lists and identify patients at a lower risk of consequences from OSA. Again, the discrepancy in the hypopnea detection led us to overestimate the severity of 3/40 patients (7.5%) who were allocated to the severe group. Two further patients (5%) were scored as normal and were false negatives. They had an AHI at Nox T3 of 17.9 and 18.4 events per hour, respectively, and a basal SaO_2_ of close to 92%, therefore near to the steep portion of the oxyhemoglobin dissociation curve. In this case, the definition of hypopnea will consider as significant any change in ventilation greater than 30%, as SpO_2_ will sharply decrease and validate the event. In these two patients, Airgo™ underestimated the severity of the disease.

Finally, a group of 16 patients had miscellaneous disorders that caused turbulence in respiratory rhythm but could not be considered OSA patients. The underlying disorders were myoclonus in 4 patients, insomnia in 6 patients, periodic breathing with central hypopneas in 2 patients, and REM-related OSA in 4 patients. Despite the heterogenous origin of the disorder, all patients had in common an instability of respiratory rhythm, caused by sighs in the myoclonus and insomnia, in periodic breathing linked to the crescendo-decrescendo pattern of ventilation, and in REM-related due to the occurrence in clusters not related to body position. All these disorders caused a sharp decrease of RICs in the target area, going from severe to normal-mild in three deciles, a trend which is not typical of the more repetitive pattern of OSA. Airgo™ identified 14 out of 16 patients correctly (87.5%) and considered normal the other 2 patients (one myoclonus and one REM-related). The opportunity to discriminate amongst these patients can be considered specific to Airgo™ and is potentially useful in clinical practice. The correct diagnosis requires a setting more sophisticated than usual home sleep polygraphy, with at least leg movement sensors for myoclonus, standard PSG for insomnia, and REM-related OSA (18). Using a standard home sleep monitor would be inconclusive, and again, this screening tool could help in sparing resources and avoiding useless polygraphies.

Considering the overall performance of the Airgo™, data on sensitivity and specificity are consistent with the opportunity to implement the use of the device as a screening tool for sleep respiratory disorders, particularly for OSA. The positive likelihood ratio is above 10 and the negative likelihood ratio is below 0.1, both suggesting reliability in ruling in and ruling out the disorder. The classification of severity in a few patients (3 normals and 2 mild OSAs) has been overestimated. This is caused by the above-mentioned difference in scoring hypopneas only when at least a 3% desaturation occurs compared to the modulation of tidal volume assessed by the algorithm of Airgo™. In the real world, this could mean a few more studies performed in the diagnostic path following the screening, but considering the data reported in Table 5, the net amount of time saved and the consequent possible increase in the number of patients diagnosed properly seem to balance these few patients who would have to undergo a polygraphy without need.

### Limitations of the Study

This is a monocentric study on patients referred to a sleep center for symptoms, clinical signs, or witnessed apneas. It is possible that in a specific subgroup of patients or in the general population sensitivity and specificity could vary. The number of patients tested is large, but the results should be validated in bigger numbers. The percentage of females is low, 23 out of 120 patients recruited, a 19% rate which does not allow separate analysis of data to assess a possible difference in sensitivity gender-related. This is not surprising since patients of sleep centers are predominantly males and it is possible that the results of the present study cannot be representative of the female population. The results have not been compared with other screening tools (i.e., questionnaires) since the study focused on the use of the device *per se* and at least two groups of patients (pOSA and non-OSA patients) are not stratified by common questionnaires. Airgo™ was positioned by sleep lab technicians and despite the simplicity of the procedure, it is possible that in less experienced centers or when the patient himself has to wear the band more studies could fail. Considering that one of our patients had switched off the device before the beginning of the study, the Airgo™ was then modified to avoid this inconvenience. Pregnancy is a condition that changes respiratory mechanics and adds a second breathing organism. Considering the characteristics of the curve detected in the validation study (11) the device would probably be unreliable. Patients with recent thoracic or abdominal surgery are poor candidates due to the effect on ventilatory mechanics of the procedure and complete recovery should be awaited. Kyphoscoliosis and other thoracic abnormalities could reduce the reliability of Airgo™, but have not been specifically tested in the present study and none of the patients enrolled had thoracic abnormalities.

### Perspectives

A promising application of the device is the possibility to perform monitoring over consecutive nights, up to 21 with a single battery. The memory of the device supports the storing of every single breath. The use of a single night study has been questioned, particularly in mild to moderate disease, posture-related OSA, and in patients with irregular sleep habits (i.e., shift workers, binge drinking on the weekend). In the prospect of personalized medicine and in the correct allocation of risk category, using the device for at least three nights could be more informative than a single-night traditional study (19). The assessment of treatment results alternative to CPAP could also benefit from this approach. Mandibular advancement devices and position trainers can change their effectiveness from night to night, a piece of information that standard sleep studies do not provide. Another group of patients who could benefit from this approach is those with OSA and insomnia, who are often disturbed by the number of sensors of usual polygraphy and who again have a high night-to-night variability. The opportunity to have a single band and more than one night of assessment could be much more informative than traditional techniques in a consistent number of patients, quantified in different studies as between 20 and 60% of the total number of OSA patients.

The use of Airgo™ as a screening tool is only a part of the rich, informative signal processing of the device. The visualization of vectors recorded overnight is far more informative, but goes beyond the aim of this study which excluded the operator’s skill in interpreting the results and just applied the automatic analysis to obtain a low-cost, time-saving screening tool.

## Conclusion

Airgo™ is a reliable tool to screen patients with respiratory sleep disorders. In OSA patients, it discriminates between severe OSA and positional OSA and is quite accurate in ruling out those who have no respiratory disturbances. Mild to moderate OSA is correctly identified as well in the large majority of the patients, but the difference in technology leads to some discrepancy in the hypopnea detection compared to standard polygraphy. A clinically useful additional information is the detection of patients who have irregularities of breathing patterns as a consequence of sleep fragmentation (i.e., insomnia, myoclonus), candidates for polysomnography for proper diagnosis, avoiding an inconclusive home sleep study, and contributing to optimize resources and the waiting list of the sleep lab.

## Data Availability Statement

The raw data supporting the conclusions of this article will be made available by the authors, without undue reservation.

## Ethics Statement

The studies involving human participants were reviewed and approved by “Salvatore Maugeri Foundation” Ethics Committee. The patients/participants provided their written informed consent to participate in this study.

## Author Contributions

AB and DK contributed to conception and design of the study. MG and FR organized the database. AB and CS scored the studies. AB, DK, and EM wrote the manuscript. All authors contributed to manuscript revision, read, and approved the submitted version.

## Conflict of Interest

ICS Maugeri holds stocks of Myair Inc. DK, CEO of both Myair Inc. and its subsidiary Myairgo Italy Srl, has a clear conflict of interest in this study. DK is a major shareholder in Myair Inc. as well as the inventor and principal developer of the Airgo technology platform and the author of all related patents. DK has provided much of the know-how and developed many of the algorithms used to produce the Airgo Sleep Reports being tested in this clinical trial. The remaining authors declare that the research was conducted in the absence of any commercial or financial relationships that could be construed as a potential conflict of interest.

## Publisher’s Note

All claims expressed in this article are solely those of the authors and do not necessarily represent those of their affiliated organizations, or those of the publisher, the editors and the reviewers. Any product that may be evaluated in this article, or claim that may be made by its manufacturer, is not guaranteed or endorsed by the publisher.
